# Reproducibility of quantitative analysis of aortic 4D flow data

**DOI:** 10.1186/1532-429X-15-S1-P126

**Published:** 2013-01-30

**Authors:** Petter Dyverfeldt, Michael D Hope, Monica Sigovan, Jarrett Wrenn, David Saloner

**Affiliations:** 1 University of California San Francisco, San Francisco, CA, USA; 2 Linköping University, Linköping, Sweden

## Background

3D cine phase-contrast CMR ("4D Flow") permits quantitative assessment of anomalous alterations of aortic blood flow. Two hemodynamic parameters that have been used for this purpose is the wall shear stress (WSS), which is known to regulate endothelial cell function, and the normalized flow displacement from the vessel center, which was recently shown to correlate with increased growth rates of ascending aortic dilation [1,2]. Analysis of these hemodynamic parameters requires that a user 1) positions a 2D plane of interest in the volumetric 4D Flow dataset and 2) delineates the contour of the vascular lumen in this 2D plane. We set out to assess the reproducibility of 4D Flow-based estimation of WSS and normalized flow displacement at these two critical levels of user-interaction. Furthermore, we assessed which of the parameters correlate best with aortic growth.

## Methods

25 patients previously studied with 4D Flow imaging were included. Previously reported data on interval aortic growth was available for each subject [2].

**Figure 1:**
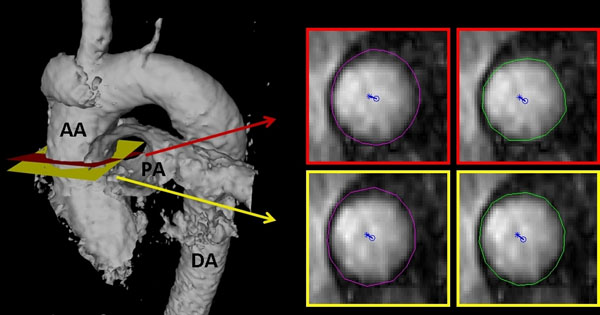
Example case demonstrating the reproducibility analysis performed in the present study. An isosurface of the thoracic aorta is provided on the left to show the location of the planes selected independently by two observers (one in red, the other in yellow). Each plane was then independently segmented by two separate observers for quantification of CMR hemodynamics parameters (one contour in purple, the other in green). Normalized flow displacement from the vessel center (blue circle) is depicted for each of the planes.

CMR velocity data from a plane perpendicular to the ascending aorta just distal to the sinotubular junction was collected independently by two blinded reviewers, and then separately segmented by two blinded observers (Figure 1). Subsequently, the following parameters were calculated: normalized flow displacement, maximum peak-systolic WSS, maximum of systole-averaged WSS, mean peak-systolic WSS, minimal peak-systolic WSS. Normalized flow displacement was calculated as in reference [1]. WSS calculation was performed with propriety software (Flow Tool) [4]. Reproducibility analysis and correlation with interval aortic growth were performed.

## Results

Inter-observer correlations with regards to plane selection and contour delineation are reported in Table 1. For the contour delineation, correlation coefficients were 0.97-0.98 for normalized displacement and 0.78-0.96 for the WSS parameters. For the plane positioning, these correlation coefficients were 0.91-0.93 for normalized displacement and 0.51-0.85 for the WSS parameters. Flow displacement best correlated with interval aortic growth (r = 0.65). The range of WSS parameters did not correlate well with growth (r < 0.15).

**Table 1:** Reproducibility analysis: inter-observer correlations

		Mean peak-systolic WSS	Max peak-systolic WSS	Min peak-systolic WSS	Max systole-averaged WSS	Min systole-averaged WSS	Normalized Flow Displacement
Observer_planes_#1 versus Observer_planes_#2	Contours #1	0.71	0.67	0.47	0.74	0.71	0.91
	
	Contours #2	0.82	0.85	0.51	0.85	0.70	0.93

							

Observer_contours_#1 versus Observer_contours_#2	Planes #1	0.93	0.87	0.84	0.87	0.88	0.98
	
	Planes #2	0.96	0.91	0.81	0.90	0.78	0.97

## Conclusions

Normalized flow displacement is a reproducible hemodynamic marker that shows good correlation with interval aortic growth. Reproducibility of contour delineation for WSS analysis was good and in line with previous reports [3]. However, markedly lower reproducibility was found for the plane positioning step of the WSS analysis. Normalized flow displacement should be considered in future work aimed at identifying and risk-stratifying patients who are likely to develop clinically significant aortic dilation based on CMR-estimated hemodynamic parameters.

## Funding

Covidien/Radiologic Society of North America Research Scholar Grant 2012-2014.
